# It's About Time: Minimizing Hardware and Software Latencies in Speech Research With Real-Time Auditory Feedback

**DOI:** 10.1044/2020_JSLHR-19-00419

**Published:** 2020-07-08

**Authors:** Kwang S. Kim, Hantao Wang, Ludo Max

**Affiliations:** a Department of Speech and Hearing Sciences, University of Washington, Seattle; b Haskins Laboratories, New Haven, CT

## Abstract

**Purpose:**

Various aspects of speech production related to auditory–motor integration and learning have been examined through auditory feedback perturbation paradigms in which participants' acoustic speech output is experimentally altered and played back via earphones/headphones “in real time.” Scientific rigor requires high precision in determining and reporting the involved hardware and software latencies. Many reports in the literature, however, are not consistent with the minimum achievable latency for a given experimental setup. Here, we focus specifically on this methodological issue associated with implementing real-time auditory feedback perturbations, and we offer concrete suggestions for increased reproducibility in this particular line of work.

**Method:**

Hardware and software latencies as well as total feedback loop latency were measured for formant perturbation studies with the Audapter software. Measurements were conducted for various audio interfaces, desktop and laptop computers, and audio drivers. An approach for lowering Audapter's software latency through nondefault parameter specification was also tested.

**Results:**

Oft-overlooked hardware-specific latencies were not negligible for some of the tested audio interfaces (adding up to 15 ms). Total feedback loop latencies (including both hardware and software latency) were also generally larger than claimed in the literature. Nondefault parameter values can improve Audapter's own processing latency without negative impact on formant tracking.

**Conclusions:**

Audio interface selection and software parameter optimization substantially affect total feedback loop latency. Thus, the actual total latency (hardware plus software) needs to be correctly measured and described in all published reports. Future speech research with “real-time” auditory feedback perturbations should increase scientific rigor by minimizing this latency.

During the last two decades, there has been a strong interest in various aspects of speech production related to auditory–motor interactions and auditory–motor learning (e.g., Cai et al., 2014, 2012; Daliri & Max, 2018; Daliri et al., 2018; Feng et al., 2018; Houde & Jordan, 1998; Keough et al., 2013; Max et al., 2003; Mollaei et al., 2016, 2013; see also Caudrelier & Rochet-Capellan, 2019, for an extensive review). Many experiments in this general area of work involve auditory feedback perturbation paradigms in which participants' acoustic speech output is experimentally altered and played back via earphones/headphones “in real time.” In an ideal setup, the perturbed auditory feedback would be provided at the same time as when it is produced. In reality, however, there will always be an inevitable delay inherent in the audio and/or computer hardware and the signal processing algorithms implemented by those hardware components. For example, in order to track the speaker's fundamental frequency or formant frequencies—and then manipulate those frequency values prior to outputting the feedback signal—the involved signal processing algorithms require a minimum window length, causing some amount of delay in the feedback output. In addition, analog-to-digital (A/D) and digital-to-analog (D/A) converters built into the audio or computer hardware add to the overall input–output latency. Although the auditory feedback in these experiments is often referred to as a “real-time” signal, certain combinations of hardware and software components might result in latencies that are sufficiently long to potentially have an impact on the experimental results.

It has been shown, for example, that auditory feedback delays disrupt aspects of ongoing speech production when the delay reaches 50 ms or more (Stuart et al., 2002). Additionally, studies on speech auditory–motor learning across trials have demonstrated that adaptation to frequency-shifted auditory feedback is reduced or eliminated when the feedback signal is delayed by 100 ms (Max & Maffett, 2015; Mitsuya et al., 2017; work currently in progress in our lab is testing the effect on adaptation of delays shorter than 100 ms). In other experiments, including those focused on measuring participants' immediate phonatory or articulatory compensation for unpredictable within-trial perturbations in the auditory feedback, participant response latency often is one of the dependent variables, and the validity of such measurements depends on determining and reporting the feedback loop's input–output latency. Thus, in speech and voice studies that provide participants with auditory feedback, there are numerous experimental situations where scientific rigor demands an accurate determination and reporting of the involved hardware and software latencies. Here, we focus specifically on methodological issues involved in studies that implement real-time auditory feedback perturbations, and we will offer concrete suggestions for increased rigor and reproducibility in this particular line of work.

Generally speaking, there are two options for an experimental setup that implements auditory feedback perturbations. The first option is to use a commercially available vocal processor (e.g., Bauer & Larson, 2003; Behroozmand et al., 2009; Feng et al., 2011; Heller Murray et al., 2019; Loucks et al., 2012; Max et al., 2003; Rochet-Capellan & Ostry, 2011). One device that has been used in speech research, although no longer manufactured now, is TC Helicon's VoiceOne, a rack-mounted digital processor that is computer controllable by means of Musical Instrument Digital Interface signals. The VoiceOne processor is able to implement independent pitch and formant shifting with a confirmed total latency of 10–11 ms when using the “live” setting (e.g., Feng et al., 2011, 2018; Jones & Keough, 2008; Keough et al., 2013; Lametti et al., 2014; Max & Maffett, 2015; Mollaei et al., 2016, 2013; Rochet-Capellan & Ostry, 2011). The second option consists of using custom-designed signal processing software with a digital signal processing board (e.g., Houde & Jordan, 2002; Purcell & Munhall, 2006; Villacorta et al., 2007) or a commercially available consumer-grade audio interface (Cai et al., 2008; Tourville et al., 2013). In the latter category—custom signal processing software used with an audio interface—the MATLAB-based application Audapter (a MEX interface built from C++ source code) has gained great popularity due to its capability to implement many different real-time perturbations (e.g., Abur et al., 2018; Ballard et al., 2018; Cai et al., 2014, 2012, 2008, 2010; Daliri & Dittman, 2019; Daliri et al., 2018; Franken, Acheson, et al., 2018; Franken, Eisner, et al., 2018; Klein et al., 2018; Lametti et al., 2018; Reilly & Pettibone, 2017; Sares et al., 2018; Sato & Shiller, 2018; Stepp et al., 2017).

Interestingly, for auditory feedback perturbations with Audapter, the overall system input–out latency has been reported to be as short as 10 ms and as long as 45 ms, but in most studies, it has been listed in the range of 10–15 ms. Unfortunately, measurement methodology issues have caused misleading information to be reported. As we demonstrate below, when the Audapter latency is claimed to be as short as 10–15 ms, the measurement refers to “only” the software processing time, and it does “not” take into account the equally important and additive latency associated with the Audio Stream Input/Output (ASIO) hardware audio interface. The relevant steps that “should” be taken into account for such a latency report include all three of (a) audio signal input to the interface (A/D conversion), (b) software signal processing, and (c) audio signal output from the interface (D/A conversion). Indeed, Audapter's own software processing latency (with default settings) already is 10–14 ms, and the input–output temporal asynchrony data that are available within the Audapter program refer exclusively to this software processing time (i.e., it reports the time delay between the digital input signal after it has already been A/D converted by the audio interface and the processed digital output signal before it is D/A converted and output by the audio interface). Thus, it is necessary to bring attention to the fact that—contrary to common assumptions—the additional hardware delays associated with the audio interface are not at all negligible (as we show below, they can be even longer than the software processing latencies) and need to be accurately taken into account and reported.

In order to truly know the total system latency that accounts for both the hardware latencies and the Audapter (or other software) latency, the only option is to simultaneously record—and then measure the temporal asynchrony between—the (a) original microphone signal “before” it is digitized by the audio interface and (b) the processed signal to the headphones “after” it has been output by the audio interface. To date, there is no published documentation of such correctly measured latencies for setups where Audapter is linked with any of the commonly used audio interfaces. In this study, we therefore investigated for two different computers and five different ASIO-compatible audio interfaces (a) the devices' own intrinsic round-trip latency (RTL), (b) the Audapter processing time, and (c) the total system input–output latency when implementing a typical formant-shift perturbation. We also tested nondefault Audapter parameter values as a strategy to optimize feedback latency. The data provide realistic measures of the overall feedback delay in typical Audapter setups and demonstrate that some audio interfaces and software options perform significantly better in terms of avoiding unwanted feedback delays.

## Method

### Instrumentation

#### Computers

We ran the Audapter software on two different computers (referred to as operating computers to distinguish them from a simultaneously used recording computer) in conjunction with the different audio interfaces in order to verify whether processing and overall latency differ depending on computing power. Given that Audapter is often used with laptop-based systems for portability, we first tested a Dell laptop (Latitude E5570) with Intel core i7-6820HQ processor, 16-GB RAM memory, and Windows 7 Professional operating system. The laptop ran on external power and in high-performance mode. The second operating computer was a Dell workstation (Precision Tower 7510) with Intel Xeon E5-2640 v4 processor, 32-GB RAM, and Windows 7 Professional operating system. During the tests, no other computer programs were open except for the Sophos antivirus program that ran in the background, as required by the University of Washington.

We used an additional Dell laptop (Dell Inspiron 14R-N4010) as the recording computer. This recording computer's stereo line input was used to record on separate channels (a) the direct microphone signal (i.e., prior to reaching the audio interface and Audapter) and (b) the final output signal (i.e., after being input to the audio interface, processed in Audapter, and output from the audio interface). This recording laptop was also connected to external power and also operated in high-performance mode. Off-line, the latency between the recorded stereo channels was measured using Praat, a free software for analyzing speech sounds (Boersma & Weenink, 2019).

#### Audio Interfaces

For real-time processing, Audapter requires an ASIO-compatible audio interface. The interface most extensively tested by the developers of Audapter, the MOTU MicroBook (Cai, 2014), is now outdated. We therefore included in our tests a more recent model of the MicroBook series, the MOTU MicroBook IIc. Another interface mentioned in the Audapter manual is the MOTU Ultralite, but this model is also outdated now. Thus, again, we included in our testing a more recent model of the Ultralite series, the MOTU Ultralite AVB. In addition, we also tested our own lab's preferred interface, the RME Babyface Pro, as well as two more popular interfaces, the Presonus Studio 192 Mobile and Tascam US 2 × 2. All tested interfaces are portable (as opposed to rack-mounted).

All audio interfaces were set up to use a sampling rate of 48 kHz and a buffer size (or frame size) of 96 or 128 samples, depending on which options were supported by the device. The MOTU MicroBook IIc and RME Babyface Pro support a buffer size of 96 samples (which is used in examples in the Audapter manual); thus, the smaller buffer size was used for these two interfaces. The other interfaces did not support a buffer size of 96 samples; only 64 and 128 samples. According to our tests, a buffer size of 64 samples is too short for the present purposes as the 1.3-ms frame length (64 buffer size/48 kHz before downsampling) resulted in unreliable formant tracking. Therefore, for those interfaces that did not support buffer size 96, we tested the next shortest buffer size of 128 samples. Given that the RME Babyface Pro supports both buffer sizes 96 and 128, an additional test was completed for this device, with the buffer size set to this greater value of 128 samples.

#### Audio Drivers and Software Setting

In addition to each manufacturer's default ASIO driver for the operating system, we also installed ASIO4ALL, a hardware-independent low-latency ASIO driver. This allowed us to test whether there is any advantage to using the manufacturers' own default drivers.

Unless specified otherwise,^1^ the Audapter software itself was used with its default parameters from getAudapterDefaultParams.m as included in the software package (Cai et al., 2008; Tourville et al., 2013). We implemented an auditory perturbation that consisted of upward shifts of Formant 1 (F1) and Formant 2 (F2). To achieve this perturbation, we set *bRatioShift* = 1 for a ratio shift, all 257 values of *pertPhi* = π/4 radians for a 45° shift in the F1-by-F2 vowel space, and all 257 values of *pertAmp* = 0.2 for a 20% shift in that direction (thus a 20% formant frequency upshift with a 45° direction in the vowel space). Consequently, both F1 and F2 received a 14% upshift in hertz, sin(π/4 radians) × 20%, which corresponds to 229 cents, 1200 × log_2_(1.14/1).

The downsampling rate parameter, *downFact*, was set to 3 (downsampling the original 48 kHz signal to 16 kHz) for most of the tests with interfaces that used a buffer size of 96 samples, as suggested in the Audapter manual (Cai, 2014). An exception was a separate test conducted to compare the effect on processing latency of setting this parameter to 3 versus 4 (i.e., increased downsampling). For interfaces with a buffer size of 128 samples, we always used *downFact* at 4 (downsampling 48–12 kHz). Audapter was operated with Audapter(“start”) and Audapter(“stop”) functions to start and stop the auditory perturbations. It was operated for 5 s before it was stopped. Audapter recordings were retrieved via the AudapterIO(“getdata”) command and saved into wav files, resampling to 44.1 kHz.

Users have the option of shortening Audapter's software processing latency by lowering the *nDelay* value. Given that only odd positive integers are accepted, we tested Audapter's processing latency with *nDelay* values of 1, 3, and 5, in addition to the default value of 7. With *nDelay* of 1, reliable formant tracking was not possible, so only the results from *nDelay* of 3 and *nDelay* of 5 are reported and compared with that of *nDelay* of 7. According to the manual (Cai, 2014), the *nDelay* parameter determines the length of the internal processing window used by Audapter (internal processing window = 2 × *nDelay* − 1) for formant tracking. Audapter then filters the (*nDelay*)th frame to perturb the formant frequencies by the desired amount (chosen by the user) and sends this altered audio signal to the output channel. Hence, in the software, the resulting temporal asynchrony between the input and output channel becomes 2 × *nDelay* − 1 − *nDelay = nDelay* − 1 frames. For the default *nDelay* value of 7, there would be a latency of six frames or 12 ms (2 ms [96 buffer / 48 kHz] × 6 frames; Cai, 2014).^2^ We emphasize again that this is software latency^3^ only, without taking account of the additional hardware latencies, and, thus, not an accurate representation of the total feedback loop delay.

It should be noted that Audapter's processing latency is determined by different parameters, depending on which type of auditory perturbation is being applied. This report tested the latencies during formant-shift perturbations, and thus, the results are only directly applicable to this type of experimental manipulation. Nevertheless, the procedures that we describe for measuring the latencies accurately and most of the resulting recommendations for minimizing those latencies are generalizable to other types of perturbations (such as pitch shifts).

### Testing Procedure

#### Hardware Latency

**Figure 1.**
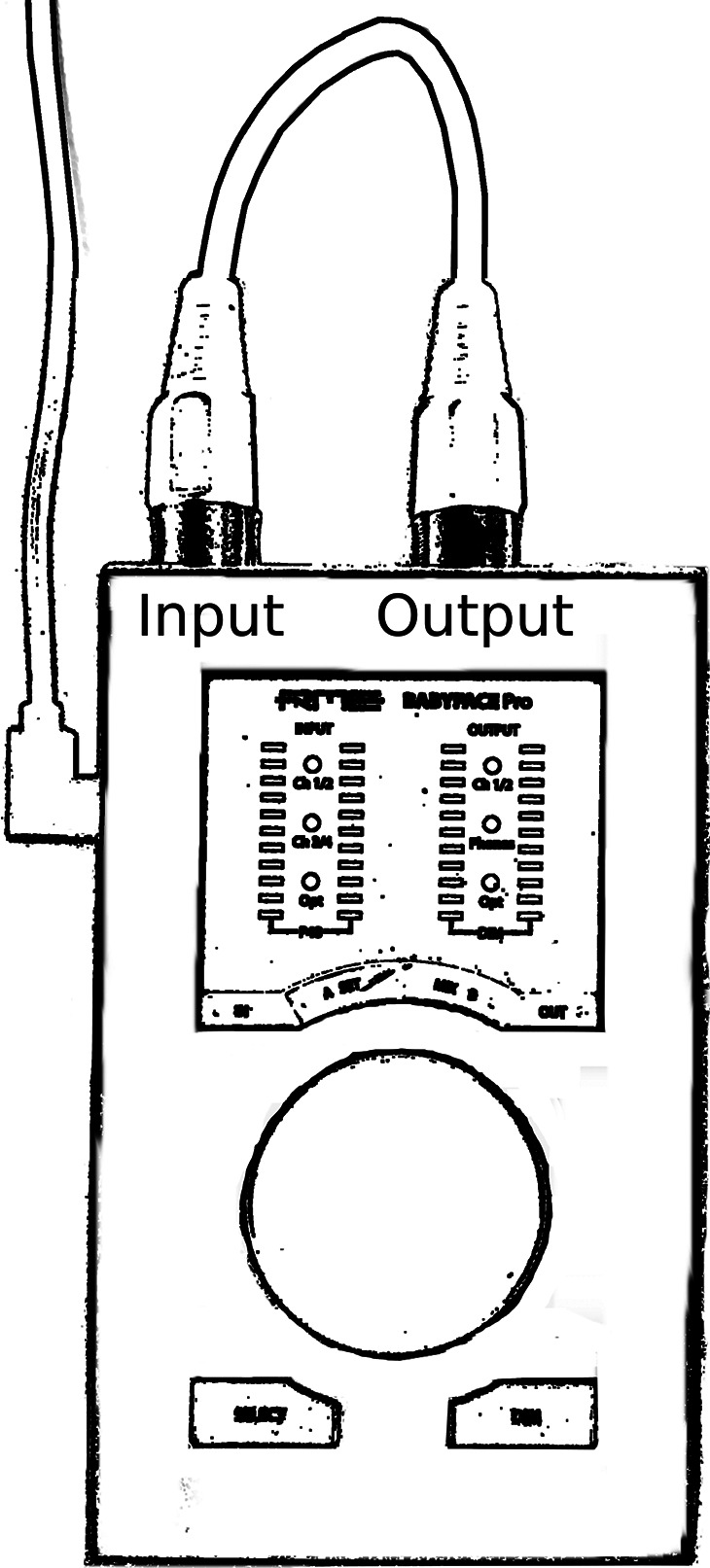
Setup for measuring hardware-only round-trip latencies. The audio interface's output was looped back to its input, and the latencies were measured with the RTL Utility software. RME Babyface Pro depicted here as an example audio interface.

We used the RTL Utility software tool (Oblique Audio, www.oblique-audio.com) to measure RTL (input latency plus output latency) of the audio interfaces. This is accomplished by looping the audio interface's output back to its own input (see Figure 1). When the RTL Utility program causes the interface to generate an audio signal, this signal goes through the output component of the interface and then comes back through the input component of interface to reach the computer. The RTL Utility measures the latency between the two events (sending and receiving the signal) to determine the RTL, as this measurement reflects the sum of the input and output latencies. We refer to this RTL also as the hardware latency. For the present tests, we ran the RTL Utility 5 times for each individual interface.

#### Total Feedback Loop Latency

**Figure 2.**
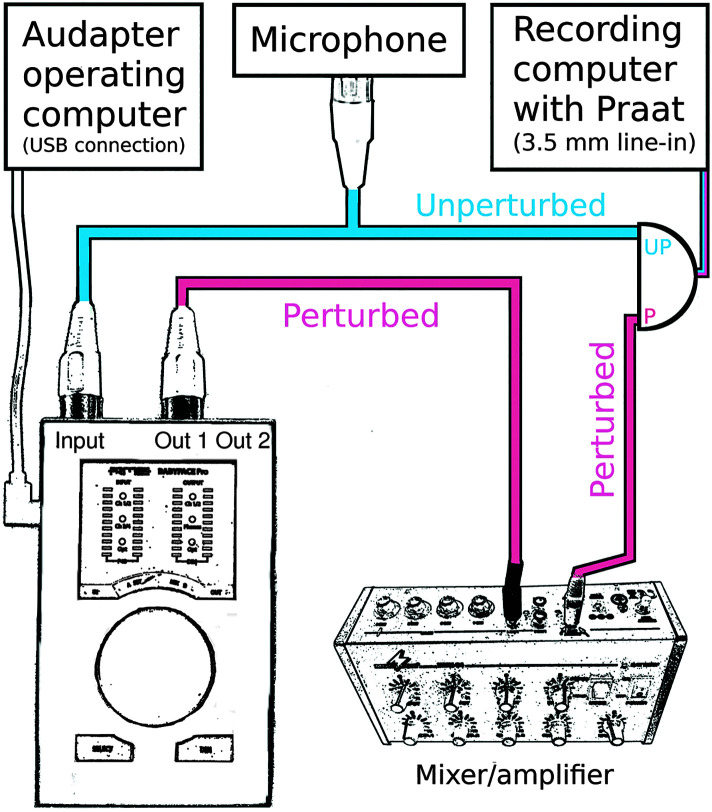
Correct setup for measuring total feedback loop latency. The unperturbed signal arrives at the recording computer without traveling to the audio interface, while the perturbed signal travels through the audio interface (input), operating computer (software processing), audio interface again (ouput), and a mixer or amplifier before arriving at the recording computer. The total latency, accounting for both hardware and software latencies, is measured.

In order to measure the “total feedback loop latency” that occurs in a speech experiment, we needed to simultaneously record the unperturbed subject ouput (i.e., the original microphone signal) and the perturbed feedback signal (i.e., the formant-shifted signal routed to the subject's earphones). To record the first of these two signals, we split the signal from the microphone (AKG C544, Shure) with an XLR splitter, such that both the tested USB audio interface and one audio channel of the recording laptop received the exact same microphone signal at the same time. To record the formant-shifted output, the signal that had been processed by Audapter and that was output by the audio interface was routed to a mixer (MMX-24, Monacor) in order to be amplified and sent to the second channel of the recording laptop's stereo line-in input (see Figure 2). The two channels of data were saved on the recording computer using the Praat software. Recordings were made while a male speaker's production of the word “tuck” was played back from a loudspeaker at least 5 times for each test. We then measured, from the Praat recordings, the temporal asynchrony between the original, unperturbed microphone signal and the perturbed feedback signal for five trials.

#### Audapter Software Processing Latency

A first, “indirect” method of determining the Audapter software processing latency was based on the fact that additional latencies due to mixer and audio cables were negligible (less than 0.05 ms). Thus, any part of the total feedback loop latency not caused by the interface hardware can be attributed to Audapter processing. Consequently, it was possible to subtract the measured hardware latencies from the overall feedback loop latencies to obtain an estimate of Audapter's signal processing time. As a second “direct” method of determining Audapter's software processing latency, we also compared the temporal asynchrony between the two channels (input and output corresponding to unperturbed and perturbed signals) stored within Audapter's own recording on the operating computer. Using Praat to analyze the recordings from five trials, we measured the time difference between the two Audapter channels. These measures of the time offset between the unperturbed and perturbed signals stored by Audapter (i.e., this second direct method) can then be compared with the estimates obtained by subtracting hardware latency from total feedback loop latency (i.e., the first indirect method).

#### Effect of Audapter's downFact Parameter

In Audapter, the default value of *downFact* at 3 downsamples the original audio input from a sampling rate of 48 kHz and a buffer size of 96 samples to a sampling rate of 16 kHz and a buffer size of 32 samples. However, some researchers have used *downFact* at 4 to downsample the original signal to a rate of 12 kHz and a buffer size of 24 samples (e.g., Daliri et al., 2018). Given that Audapter does allow users to specify this amount of downsampling in order to lower computational load, we tested whether or not the amount of downsampling affects processing latency. For this purpose, we used the RME Babyface Pro audio interface, and both the laptop and desktop operating computers with *downFact* was set to either 3 or 4.

#### Effect of Audapter's nDelay Parameter

As mentioned above, we also aimed to examine whether Audapter's processing time can be shortened by lowering the *nDelay* parameter from its default value of 7 to 5 or 3. Caution is warranted, however, given that a shorter processing window could degrade the accuracy of the formant tracking and, thus, also the intended formant perturbation. We therefore completed a more extensive test to verify formant-tracking accuracy for each of the three *nDelay* values 7, 5, and 3. For this purpose, we used a data set consisting of 30 repetitions of the words “tech,” “tuck,” and “talk” spoken by an adult male speaker. The of 90 trials was played back 3 times at identical volume from a computer loudspeaker (AX210, Dell) into a microphone (SM58, Shure) connected to the above described RME Babyface Pro audio interface. For each run of 90 trials, Audapter processed the incoming signal and tracked the formants at a given *nDelay* value. Off-line, F1 and F2 were calculated for each trial as the average value across the middle 40%–60% of the corresponding formant track.

#### Effect of Windows Operating System Version Installed on the Operating Computer

As mentioned above, testing was generally completed with both the laptop and desktop operating computers running Audapter software within the Windows 7 Professional operating system. However, given that Microsoft will no longer provide support for Windows 7 starting in 2020, we decided to also do a test of Windows 7 versus Windows 10 on one of these operating computers. Therefore, after completing all the above described tests, we upgraded the Dell Latitude E5570 machine's operating system to Windows 10 Enterprise LTSC (Long-Term Service Channel), and we repeated a set of total latency measurements with the RME Babyface Pro interface and the Audapter parameter *downFact* set to 3. As we had done in Windows 7, we ran this set of tests again with each of the three *nDelay* values 7, 5, and 3.

## Results

### Hardware Latency

Hardware-related RTLs for all tested combinations of audio interfaces and operating computers are listed in Table 1. First, it is clear that the use of the ASIO4ALL driver should be avoided. In all cases, the use of the audio interface manufacturer's own driver resulted in much shorter hardware latencies than the ASIO4ALL driver. For example, the two fastest devices (RME Babyface Pro and MOTU Ultralite AVB with the HSO [Host Safety Offset] setting at 16) showed hardware latencies that were approximately 8 ms shorter when operated with the default driver. In addition, hardware latency for the fastest device (RME Babyface Pro) was much more consistent with the default driver (the standard deviation across repeated runs with a buffer size of 96 samples was 0.001 ms) than with the ASIO4ALL driver (*SD* > 1.5 ms). Both results suggest significant compatibility issues between the ASIO4ALL driver and some audio interfaces. We will therefore focus our remaining description of the test results on those obtained with the manufacturers' own drivers.

**Table 1.** Hardware-only round-trip latencies (RTLs; in milliseconds), measured with the RTL Utility (setup shown in Figure 1), for all combinations of audio interface and operating computer.

Audio interface/computer	Hardware RTL with manufacturer ASIO driver	Hardware RTL with ASIO4ALL driver
Devices with a buffer size of 96 samples		
MOTU MicroBook IIc/Latitude E5570	13.081 ± 0.002	14.082 ± 0.000
RME Babyface Pro/Latitude E5570	5.299 ± 0.001	11.287 ± 1.528
RME Babyface Pro/Precision 7510	5.307 ± 0.001	11.070 ± 1.552
Devices with a buffer size of 128 samples		
MOTU Ultralite AVB (HSO 16)/Latitude E5570	9.546 ± 0.065	17.517 ± 0.056
MOTU Ultralite AVB (HSO 16)/Precision 7510	9.544 ± 0.066	17.492 ± 0.010
MOTU Ultralite AVB (HSO 128)/Latitude E5570	14.196 ± 0.056	22.138 ± 0.001
MOTU Ultralite AVB (HSO 128)/Precision 7510	14.177 ± 0.060	22.169 ± 0.006
Presonus Studio 192 Mobile/Latitude E5570	12.550 ± 0.065	16.130 ± 0.000
Presonus Studio 192 Mobile/Precision 7510	12.472 ± 0.011	16.126 ± 0.065
RME Babyface Pro/Latitude E5570	6.648 ± 0.001	30.570 ± 11.738
RME Babyface Pro/Precision 7510	6.645 ± 0.005	13.748 ± 0.895
Tascam US 2 × 2/Latitude E5570	13.018 ± 0.002	19.488 ± 0.000
Tascam US 2 × 2/Precision 7510	12.890 ± 0.213	19.042 ± 0.373

*Note.* All tests completed with the original sampling rate of 48 kHz and separate runs for the manufacturer's ASIO driver and ASIO4ALL driver. Note that the MOTU Ultralite AVB has a Host Safety Offset (HSO) setting that influences the latency; hence, we tested this device at both 16 and 128 HSO. Means ± 1 *SD*.

It is also clear from Table 1 that the RME Babyface Pro outperformed all other tested interfaces. The Babyface Pro had the shortest latency regardless of whether it was tested with a buffer size of 96 or 128 samples and regardless of which operating computer was used. The advantage of this RME Babyface Pro over all other interfaces is substantial and not only due to its ability to use a smaller buffer size (96 vs. 128 samples). Even when tested with the same buffer size of 128 used by most other interfaces, the RME Babyface Pro was still approximately 30% faster than the second best performer, the MOTU Ultralite AVB (6.65 ms vs. 9.54 ms). When leveraging the RME Babyface Pro's ability to operate with a buffer size of 96, its latency advantage over the second best performer was as much as approximately 45% (5.30 ms vs. 9.54 ms). For all devices, hardware latency was essentially the same when tested with the laptop operating computer and the more powerful workstation desktop computer. Lastly, it is noteworthy that the MOTU Ultralite AVB dropped from second fastest to slowest when the HSO setting was at 128 (14.2 ms).

### Total Feedback Loop Latency

The middle column in Table 2 lists the total feedback loop latencies (i.e., hardware latency plus Audapter signal processing latency) as measured with the procedure displayed in Figure 2. Given the above reported finding that the manufacturers' drivers performed better than ASIO4ALL, Table 2 includes only total feedback loop latencies obtained with the manufacturers' own drivers (except for the Tascam US 2 × 2 interface whose driver was not compatible with Audapter). As expected based on our finding that hardware latencies (see Table 1) were shorter for devices with a buffer size of 96 samples (and in fact the same was true also for software latencies discussed below and listed in Table 3), total feedback loop latencies were better for devices with a buffer size of 96 samples.

**Table 2.** Total feedback loop latencies (TFLLs) comprising both hardware round-trip latency (RTL) and Audapter signal processing latency (setup shown in Figure 2) together with estimated Audapter software latencies (TFLL minus hardware RTL; in milliseconds).

Audio interface/computer	TFLL	TFLL minus hardware RTL (estimated software latency)
Devices with a buffer size of 96 samples, applied downsampling factor = 3
MOTU MicroBook IIc/Latitude E5570	27.214 ± 0.099	14.133
RME Babyface Pro/Latitude E5570	19.382 ± 0.062	14.083
RME Babyface Pro/Precision 7510	19.302 ± 0.121	13.995
Devices with a buffer size of 128 samples, applied downsampling factor = 4
MOTU Ultralite AVB (HSO 16)/Latiude E5570	28.496 ± 0.095	18.950
MOTU Ultralite AVB (HSO 16)/Precision 7510	28.473 ± 0.105	18.929
MOTU Ultralite AVB (HSO 128)/Latiude E5570	33.093 ± 0.080	18.897
MOTU Ultralite AVB (HSO 128)/Precision 7510	33.040 ± 0.141	18.863
Presonus Studio 192 Mobile/Latitude E5570	31.353 ± 0.125	18.803
Presonus Studio 192 Mobile/Precision 7510	31.389 ± 0.143	18.917
RME Babyface Pro/Latitude E5570	25.509 ± 0.098	18.861
RME Babyface Pro/Precision 7510	25.484 ± 0.048	18.839
Tascam US 2 × 2 (ASIO4ALL)/Latitude E5570	38.972 ± 0.204	19.484
Tascam US 2 × 2 (ASIO4ALL)/Precision 7510	38.567 ± 0.079	19.525

*Note.* All tests completed with an original sampling rate of 48 kHz and with each manufacturer's default driver (except for one device where, as indicated in the table, the ASIO4ALL driver had to be used due to an incompatibility between the manufacturer driver and Audapter). Means ± 1 *SD*.

**Table 3.** Audapter processing latencies (in milliseconds) quantified by using Praat software to measure the temporal difference between the unperturbed and perturbed channels recorded within Audapter.

Audio interface/computer	Audapter latency with manufacturer ASIO driver	Audapter latency with ASIO4ALL driver
Devices with a buffer size of 96 samples, applied downsampling factor = 3
MOTU MicroBook IIc/Latitude E5570	12.017 ± 0.073	11.990 ± 0.043
RME Babyface Pro/Latitude E5570	11.923 ± 0.123	11.918 ± 0.080
RME Babyface Pro/Precision 7510	11.977 ± 0.031	12.024 ± 0.037
Devices with a buffer size of 128 samples, applied downsampling factor = 4
MOTU Ultralite AVB (HSO 16)/Latiude E5570	16.000 ± 0.046	15.974 ± 0.052
MOTU Ultralite AVB (HSO 16)/Precision 7510	16.040 ± 0.037	16.025 ± 0.056
MOTU Ultralite AVB (HSO 128)/Latiude E5570	15.984 ± 0.040	15.991 ± 0.074
MOTU Ultralite AVB (HSO 128)/Precision 7510	16.020 ± 0.051	16.022 ± 0.054
Presonus Studio 192 Mobile/Latitude E5570	15.964 ± 0.030	15.993 ± 0.050
Presonus Studio 192 Mobile/Precision 7510	15.988 ± 0.056	16.022 ± 0.066
RME Babyface Pro/Latitude E5570	15.987 ± 0.033	15.986 ± 0.041
RME Babyface Pro/Precision 7510	15.974 ± 0.057	16.013 ± 0.030
Tascam US 2 × 2/Latitude E5570	N/A	16.018 ± 0.029
Tascam US 2 × 2/Precision 7510	N/A	16.011 ± 0.051

*Note.* Measures reflect software processing latency only. All tests completed with an original sampling rate of 48 kHz and separate runs for the manufacturer's ASIO driver and ASIO4ALL driver. N/A indicates that the particular interface did not function correctly with Audapter when using the manufacturer's driver. Means ± 1 *SD*.

The most important observation in these results is that the total feedback loop latencies observed in this study (from approximately 19 ms with the fastest device to approximately 39 ms with the slowest device; approximately 27 ms for the popular MOTU MicroBook IIc device) are substantially larger than claimed in most published work in which the Audapter software was used (as mentioned in the introduction, the vast majority of published studies have claimed a feedback delay of only 10-15 ms). It seems clear that the previously reported latencies took into account only the software-based signal processing latency (which is the only latency accounted for when comparing the unperturbed and perturbed signals stored by the Audapter software) while ignoring the sometimes substantial additional delays associated with the audio interface's signal input and output components.

With the appropriate measurement protocol, the overall best total feedback loop latency (19.3 ms) was observed when using the RME Babyface Pro with a buffer size of 96 samples. The second best performance (25.5 ms) was observed when the same device was used with a buffer size of 128 samples.

### Audapter Software Processing Latency (Indirect Method)

The right-side column of Table 2 summarizes the estimates of Audapter software processing latency that were obtained by subtracting hardware latencies from overall latencies. Results suggest that the software processing latency was approximately 14 ms for a buffer size of 96 samples and approximately 18.9 ms for a buffer size of 128 samples. Interestingly, both these values are slightly larger (2 ms in the case of the buffer size of 96 and 2.7 ms for buffer size of 128, values corresponding to the respective frame sizes) than what was expected based on the Audapter manual (Cai, 2014) and the results of the direct method discussed below.

### Audapter Software Processing Latency (Direct Method)

Table 3 includes results for Audapter software processing latency when measured directly as the temporal offset between the unperturbed and perturbed channels stored by the Audapter software itself. Our measurements of this offset are very consistent: 12 ms for the tests with a buffer size of 96 samples (for which the downsampling factor was set to 3) and 16 ms for the tests with a buffer size of 128 samples (for which the downsampling factor was set to 4).

These estimates closely match the processing latencies that one would predict based on the algorithm information provided in the Audapter manual (Cai, 2014). For a buffer size of 96, we predicted a processing latency of 12 ms (2 ms [96 buffer/48 kHz] × 6 input frames per input frame = 12 ms). For a buffer size of 128, we predicted a processing latency of 16.2 ms (2.7 ms [128 buffer/48 kHz] × 6 input frames = 16.2 ms). Importantly, the shorter processing latency for a buffer size of 96 versus 128 again gives an advantage to the RME Babyface Pro device. Although processing latency was essentially the same for the MOTU Microbook IIc, the latter interface's hardware in/out latency (see Table 1) was more than twice as long as that of the Babyface Pro.

As one would expect, Audapter's software processing latency was not affected by selecting the manufacturer versus ASIO4ALL driver. Similarly, it was not affected by the use of any specific audio interface (when compared across interfaces operating with the same buffer size). And lastly, based on the two operating computers used in our testing, running Audapter on a more powerful computer (with faster processor and more RAM) also did not affect software processing latency.

### Effect of Audapter's Parameter downFact

Audapter offers the option of downsampling the input signal before processing in order to lower computational load. Results for our comparison of latencies obtained with *downFact* at 3 versus at 4 are listed in Table 4. The total feedback loop latency did not improve with more downsampling, and this was true for both the laptop and desktop workstation operating computers.

**Table 4.** Total feedback loop latencies (comprising hardware latency and Audapter signal processing latency; in milliseconds) for the RME Babyface Pro audio interface with two different downsampling factors in Audapter.

Audio interface/computer	Total feedback loop latency with *downFact* of 3	Total feedback loop latency with *downFact* of 4
RME Babyface Pro/Latitude E5570	19.382 ± 0.062	19.418 ± 0.181
RME Babyface Pro/Precision Tower 7510	19.302 ± 0.121	19.462 ± 0.182

*Note.* All tests completed with an original sampling rate of 48 kHz and with the manufacturer's default driver. Means ± 1 *SD*.

### Effect of Audapter's Parameter nDelay

We tested whether reducing Audapter's *nDelay* parameter from its default value of 7 to either 5 or 3 can be used as a way to further minimize software processing latency (and thus also total feedback loop latency) without sacrificing accuracy of the formant shift perturbation. Table 5 shows that, in comparison with the default setting, an additional 4-ms processing time can be saved by switching to *nDelay* of 5, and 8-ms processing time can be saved with *nDelay* of 3. With the latter value, Audapter processing latency was reduced from 14 or 12 ms (for the direct and indirect measurement methods, respectively) to only 6 or 4 ms. Used in combination with the RME Babyface Pro hardware, this strategy is capable of reducing the total feedback loop latency from 19.4 to 11.4 ms—thereby achieving a total feedback delay as short as that provided by the commercially produced VoiceOne processor (see above).

**Table 5.** Effect of different values for the Audapter parameter *nDelay* on total feedback loop latency and Audapter's own software processing latency (in milliseconds).

Measurements	*nDelay* 7	*nDelay* 5	*nDelay* 3
Total feedback loop latency	19.384 ± 0.092	15.432 ± 0.010	11.373 ± 0.107
Audapter processing latency (indirect method)	14.084	10.133	6.074
Audapter processing latency (direct method)	12.003 ± 0.041	7.995 ± 0.025	4.006 ± 0.007

*Note.* All measurements were completed with the RME Babyface Pro audio interface, its own manufacturer-issued software driver, an original sampling rate of 48 kHz, and Audapter *downFact* of 3. Means ± 1 *SD*.

**Figure 3.**
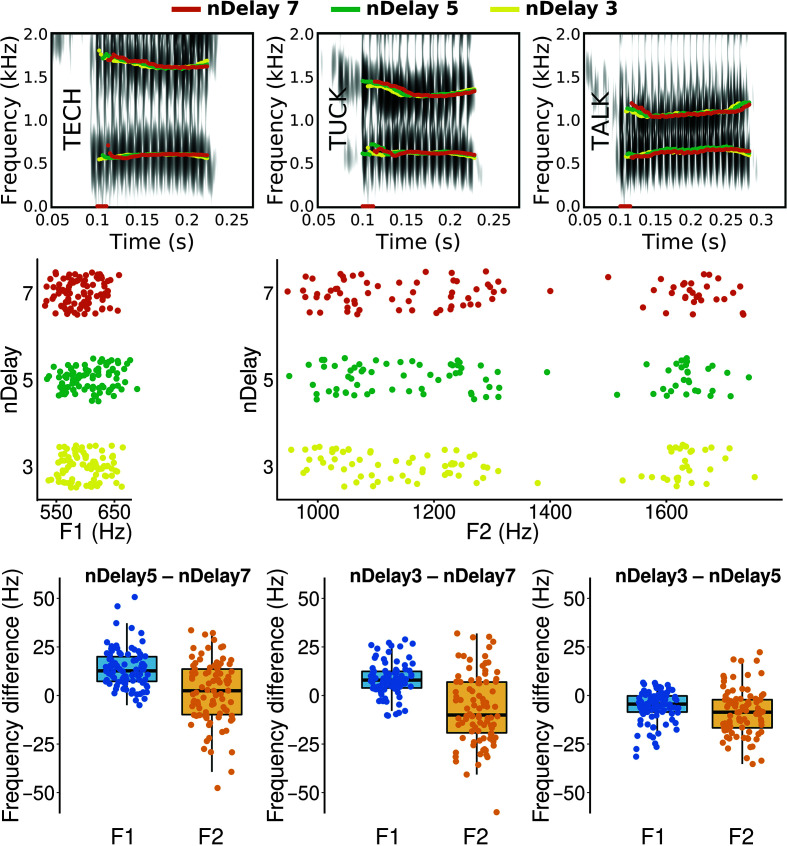
Effect on formant tracking of different values for the Audapter parameter *nDelay*. Top row: Spectrograms of a male speaker's productions of the test words “tech,” “tuck,” and “talk” with overlaid Formant 1 (F1) and Formant (F2) tracks as calculated by Audapter with the three *nDelay* values. Middle row: Audapter's F1 (left) and F2 (right) values for all 90 individual trials analyzed with the three *nDelay* values (within each trial, F1 and F2 were calculated as the average value across the middle 40%–60% of the corresponding formant track). Bottom row: Individual trial differences in formant estimates for pairs of the three analysis conditions.

In theory, the short processing window resulting from setting *nDelay* as low as 3 could degrade the accuracy of Audapter's formant tracking (thus affecting any applied formant perturbation). We therefore verified formant tracking accuracy for each of the three *nDelay* values of 7, 5, and 3. Figure 3 shows example spectrograms with overlaid formant tracks for each *nDelay* setting (top row), each individual trial's F1 and F2 values determined with each *nDelay* setting (middle row), and trial-by-trial differences in these formant values across the different *nDelay* settings (bottom row). Small differences in formant tracking can be observed, most notably when comparing the different measures on a per-trial basis (see bottom row of Figure 3). For example, *nDelay* of 5 was associated with slightly higher F1 values, especially in comparison with *nDelay* of 7. However, formant tracking for the entire data set of 90 trials was generally very similar across the different *nDelay* parameters, and there is no indication that results for *nDelay* of 3 are inferior to those for *nDelay* of 7.

### Effect of Windows Operating System Version Installed on the Operating Computer

Lastly, we examined whether different latencies are obtained when running Audapter within the newer Windows 10 operating system as opposed to the Windows 7 operating system used for all tests reported above. A direct comparison between latencies obtained with the two different operating systems installed on the same Dell Latitude E5570 operating computer is presented in Table 6. The total feedback loop latencies obtained when running Audapter with each of the three different *nDelay* values did not differ between the two operating systems.

**Table 6.** Effect of different operating systems (Windows 7 Professional vs. Windows 10 Enterprise LTSC) on total feedback loop latency.

Measurements	*nDelay* 7	*nDelay* 5	*nDelay* 3
Windows 7	19.384 ± 0.092	15.432 ± 0.010	11.373 ± 0.107
Windows 10	19.381 ± 0.058	15.446 ± 0.062	11.431 ± 0.049

*Note.* All measurements were completed with the Dell Latitude E5570 computer, the RME Babyface Pro audio interface, its own manufacturer-issued software driver, an original sampling rate of 48 kHz, and Audapter *downFact* of 3. Means ± 1 *SD*.

## Discussion

An increasing body of work on laryngeal and supralaryngeal aspects of speech production makes use of “real-time” auditory feedback perturbations. To date, little attention has been paid to quantifying the hardware and software latencies involved in the instrumentation setup for such experiments. Quantifying these latencies is critical, however, as it is known that delays in the auditory feedback loop may affect both online control and auditory–motor learning of speech (Max & Maffett, 2015; Mitsuya et al., 2017; Stuart et al., 2002). Here, we investigated hardware latency, software processing latency, total feedback loop latency, and software parameter optimization strategies for a widely used setup combining the Audapter software package (Cai, 2014) with different brands and models of commercially available audio interfaces.

First, it is clear from our tests that the choice of software driver is important. In all cases, use of the audio interface manufacturer's own proprietary driver resulted in shorter and more consistent latencies as compared with the generic ASIO4ALL driver. For our fastest audio interface, the RME Babyface Pro, using its default drivers as opposed to the ASIO4ALL driver, eliminated more than 5 ms of latency. Consequently, at least for the audio interfaces tested in this study, each interface's proprietary driver should be used for all speech experiments.

Second, despite this being ignored in most published work to date, there are substantial delays associated with the input and output components of the audio interfaces themselves, even in the absence of any software processing. In our tests with the manufacturers' faster proprietary drivers, these hardware-induced latencies ranged between approximately 5 ms and approximately 15 ms across the various audio interfaces included here. Our results very closely match the latencies reported in online forums for and by audio gear enthusiasts. For example, our 5.299-ms latency for the RME Babyface Pro interface operated with a buffer size of 96 samples at 48 kHz is very similar to individual user reports available online (ModMeister, 2018). It needs to be kept in mind that, in speech experiments, these hardware latencies additively combine with software processing latencies. Thus, the additional hardware-specific latency of 5–15 ms cannot be considered negligible. To the contrary, it should become standard practice for all auditory feedback perturbation experiments to report the “total feedback loop latency” that was in effect during data collection. Only with the latter information available can readers make informed decisions about the potential impact of the auditory feedback delay experienced by the participants.

**Figure 4.**
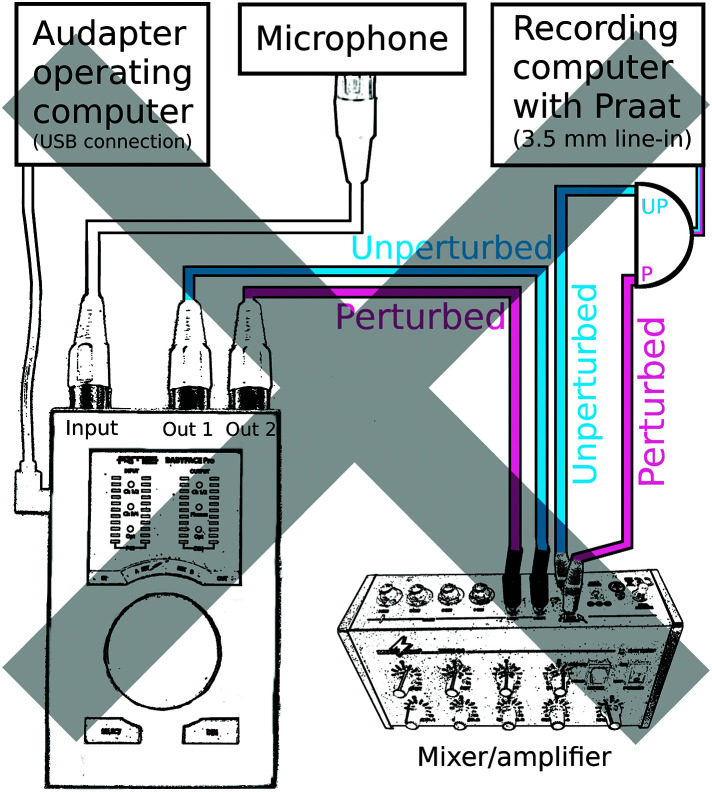
Incorrect setup for measuring total feedback loop latency. Both the unperturbed and perturbed signals arrive at the recording computer after traveling to the audio interface. As a result, the latency measured by the recording computer ignores the hardware-specific latency.

Third, to promote widespread efforts toward this goal, we have described and illustrated above how the “total feedback loop latency” can be determined by comparing the time difference between a processed signal output from the audio interface (i.e., the perturbed signal) and the original unprocessed microphone signal (i.e., the unperturbed signal). With default Audapter software settings (options for further optimization of these software settings are discussed below), our measurements of total feedback loop latency ranged from 19 to 39 ms across the different interfaces. These delays are considerably longer than most of the latencies previously reported in publications based on speech experiments with Audapter. Indeed, it seems clear that many prior reports of feedback latency in speech experiments with Audapter failed to capture the additional hardware-specific latency. This may happen if researchers compare the perturbed channel with an unperturbed channel that is also taken from the output of the audio interface (see Figure 4) rather than directly from the microphone. In that case, both channels include the audio interface's hardware latency (because even the unperturbed channel was first A/D converted by the interface, passed to the computer, and then received back from the computer), and the temporal difference between these channels does “not” reflect the true latency between a human subject's speech output and the altered auditory feedback signal. Instead, it reflects only the software latency and, thus, underestimates the total latency. Hence, we emphasize here once again the importance of measuring “total feedback loop latency,” as shown in Figure 2.^4^

Fourth, comparing the various audio interfaces tested here, we found that the RME Babyface Pro has the shortest latency. In addition, this RME Babyface Pro supports a buffer size of 96 samples, which shortens Audapter's software processing latency as compared with situations in which a buffer size of 128 samples needs to be used. Because of these advantages, our formant-shift tests showed a total feedback loop latency of 19.4 ms when using the RME Babyface Pro with Audapter (assuming default settings; down to 11.4 ms with parameter optimization as described below). In contrast, when using Audapter with some of the other interfaces, latencies approached 30 ms or, when the ASIO4ALL driver was used, even 40 ms. It has been reported that a delay greater than 25 ms can already slow down the speech rate and a delay greater than 50 ms may induce disfluencies (Stuart et al., 2002). In addition, our ongoing work suggests that auditory–motor learning in clinical populations may be differentially affected by feedback latency. It is therefore critical to run Audapter with an audio interface that has a short intrinsic latency (i.e., RTL).

MOTU Ultralite AVB with the HSO setting of 16 had the second shortest latency after RME Babyface Pro. However, whether or not this HSO setting affects Audapter's performance is not known and must be examined. The manufacturer states that the host software may experience performance issues when the parameter is set too low. Hence, it is possible that the low HSO setting may compromise the quality of auditory feedback perturbations due to formant mistracking. We recommend that researchers interested in using this particular interface test it in depth before using it with the Audapter software. Furthermore, although it is theoretically possible to shorten the hardware latency by using rack-mounted systems (e.g., RME Fireface UFX) or internal PCI-e cards (e.g., RME HDSPe AES or Lynx AES 16e/Aurora 16), various databases informing on the RTL of different interfaces indicate that any additional improvement would be marginal (i.e., < 1 ms) as compared with the RME Babyface Pro tested here (Tafkat, 2018).

Fifth, our results suggest caution when estimating Audapter's own signal processing latency. In the absence of additional latencies beyond the audio interface and Audapter software, this signal processing latency can be estimated by subtracting the hardware latency from the total latency. In our tests, this indirect estimate always slightly exceeded the latency measure obtained by measuring the temporal offset between the unperturbed and perturbed channels saved by Audapter and the prediction based on the software's *nDelay* parameter. Although the reasons for this discrepancy remain unknown, we strongly advise against using Audapter's own recordings for latency measures. They certainly do not take account of the audio interface's intrinsic latency and may not even capture the software processing latency accurately.

Sixth, in terms of trying to further optimize performance, we found that operating the interfaces and software with a more powerful computer did not yield an actual benefit. There was no difference in the total feedback loop latency across the tested operating machines (Dell Latitude laptop vs. Dell Precision desktop). Similarly, upgrading the Dell Latitude laptop's operating system from Windows 7 Professional to Windows 10 Enterprise LTSC did not affect total feedback loop latency. We also did not see any improvement in total latency when using a higher downsampling rate (e.g., Audapter parameter *downFact*) as a strategy to lower computational load as suggested in the Audapter manual (Cai, 2014). It is possible, of course, that limitations associated with the older computer technology that was available when Audapter was initially developed caused higher downsampling rates to offer significant improvements. However, most computers in use today should be adequately powerful for this aspect to be a nonissue. In contrast, our tests showed that lowering Audapter's *nDelay* parameter from the default value of 7 to 3 did substantially shorten the software processing latency. Specifically, this latency improved by 8 ms as compared with the default setting. As a result, this test situation where we implemented our formant-shift perturbation by using the RME Babyface Pro interface in combination with Audapter *nDelay* of 3 was the only instance in which we were able to achieve a total feedback loop latency of only 11.4 ms. Thus, shortening Audapter's processing latency by lowering the *nDelay* parameter is recommended.

## Concluding Remarks

Audapter is a software package that is considered to be the most comprehensive and flexible tool for perturbing auditory feedback. Most likely, it will continue to be heavily used in future studies of voice and speech motor control (Tourville et al., 2013). It is crucial, however, for researchers to be cognizant of the fact that the reproducibility of results from such studies depends on accurately measuring and reporting the overall feedback loop latency that was in effect during the experiment. As described above, this reported latency should take account of both the audio interface hardware latency and the software signal processing latency. In addition, researchers should be aware of currently available options to minimize overall feedback delays through the selection of short-latency hardware (e.g., RME Babyface Pro audio interface) and software parameters (e.g., Audapter's *nDelay*).

Undoubtedly, additional means of optimizing hardware and software components need to be investigated. For example, new technologies are available for the communication between audio interfaces and the operating computer (USB-C, Thunderbolt 3, etc.). In fact, in our tests, the Presonus 192 Studio Mobile interface used a USB 3.0 connection, but it was considerably slower than the RME Babyface Pro interface, which used a USB 2.0 connection. Nevertheless, as manufacturers introduce interfaces with more advanced connections, the effect on device latency should be examined. Another potential influence may relate to the use of Windows versus Mac versus Linux as the computer operating system (depending on availability of native drivers for the audio interface), and it should be recalled that all tests reported here were conducted only with Windows computers.

Lastly, different types of perturbation in Audapter depend on different parameters, and each of these parameters may have its own specific effect on processing delay. For example, analogous to our above manipulation of the *nDelay* parameter to shorten latency in formant-shift experiments, the *delayFrames* parameter could be used to shorten latency for pitch-shift perturbations (Cai, 2014). Investigating all additional perturbations that are possible with Audapter (e.g., pitch-shift, delay, time warping) was well beyond the scope of this work, but researchers using those feedback manipulations are strongly encouraged to apply the measurements described in this work in order to report accurate feedback loop latencies.

## Footnotes

1 In a later section, we describe how the *nDelay* parameter was also deliberately manipulated in order to test Audapter's formant shift capability with a shorter processing delay.

2 This assumes an original sampling rate of 48 kHz and a buffer size of 96 samples, making each frame size 2 ms.

3 A distinction could be made between latency that is due to software design (e.g., placing samples in a buffer) versus actual processing (e.g., doing spectral calculations and manipulations, which could depend on computer processor speed and the specific type of signal manipulation). However, results presented later in this article demonstrate that, in Audapter, the latency determined by *nDelay* encompasses any separate processing latency. Hence, we use the terms “total software latency” and “software processing latency” interchangeably.

4 Of course, if a given auditory feedback perturbation system has “additional” delay before the perturbation is actually implemented after onset of the feedback signal, then researchers should report both the signal latency as discussed in this article and the additional latency until the perturbation effectively appears in that signal. In Audapter, even the first sample of the feedback signal has gone through all processing steps. Thus, there is no additional perturbation latency beyond the feedback signal latency.
